# Comment on "Assessing Chat Generative Pretrained Transformer-4's clinical utility in acute compartment syndrome: a comprehensive evaluation of accuracy, completeness, and readability"

**DOI:** 10.1590/1806-9282.20260355

**Published:** 2026-07-17

**Authors:** Mesut Engin, Ismail Sivri

**Affiliations:** 1 University of Health Sciences, Bursa Yuksek Ihtisas Training and Research Hospital, Department of Cardiovascular Surgery – Bursa, Türkiye.; 2 Kocaeli University, Umuttepe Campus, Faculty of Medicine, Department of Anatomy – İzmit, Türkiye.

Dear Editor,

We read with great interest the article by Kılınç and Demirtaş, which provides a comprehensive evaluation of the clinical utility of ChatGPT-4 in the diagnosis of acute compartment syndrome. The authors systematically assessed the model's performance using 60 specific queries derived from the American Academy of Orthopaedic Surgeons (AAOS) 2019 clinical practice guidelines. Through a dual-specialist review process using validated instruments such as the DISCERN tool and Flesch-Kincaid Reading Ease scores, the study reports high levels of accuracy and completeness in the artificial intelligence (AI)-generated responses, alongside a notable identification of "very difficult" readability levels. This research makes a significant contribution to understanding how large language models process complex orthopedic emergencies and highlights the current balance between information quality and accessibility in AI-assisted medical consultation^1^.

However, despite the study's comprehensive evaluation, the methodology appears to overlook the critical role of prompt engineering, which is fundamental to the performance of generative AI models. The authors used a "zero-shot" approach, presenting queries directly from the AAOS guidelines without specifying a system role or providing contextual constraints for the model. Prior work has shown that simple prompt engineering strategies, such as being clear and direct about the task, assigning a specific role to the model, and controlling the output format, can substantially improve the clarity, consistency, and usability of generated content^2^.

The authors reported a mean Flesch-Kincaid Reading Ease score of 22.19, indicating that the responses were "very difficult" and potentially inaccessible to a broad audience. Prior research has consistently demonstrated that the readability of Large Language Model (LLM) outputs can be precisely modulated through strategic prompting. By incorporating specific instructions such as "explain to a medical layperson" or "provide information at a 6th-grade reading level," generative models can produce significantly more readable content compared to non-prompted or "zero-shot" approaches^3,4^.

In conclusion, this letter underscores a critical need to transition from "zero-shot" evaluations toward structured prompt engineering frameworks. Future research should examine whether these models can provide high-quality medical advice tailored to the specific linguistic needs of the user. Such refinement remains essential to bridging the gap between sophisticated AI knowledge and its practical, real-world utility in emergency orthopedic care.

## DECLARATION OF GENERATIVE AI

During the preparation of this work, the author(s) used Google Gemini 3 Pro to improve language clarity and readability. After using this tool, the author(s) reviewed and edited the content as needed and took full responsibility for the final version of the manuscript.
